# Intracranial bleeding and associated outcomes in atrial fibrillation patients undergoing percutaneous left atrial appendage occlusion: Insights from National Inpatient Sample 2016–2020

**DOI:** 10.1016/j.hroo.2023.06.002

**Published:** 2023-06-08

**Authors:** Muhammad Zia Khan, Islam Shatla, Douglas Darden, Joseph Neely, Tanveer Mir, Zain Ul Abideen Asad, Siddharth Agarwal, Sameer Raina, Sudarshan Balla, Gagan D. Singh, Uma Srivatsa, Muhammad Bilal Munir

**Affiliations:** ∗ Division of Cardiology, West Virginia University Heart and Vascular Institute, Morgantown, West Virginia; † Department of Internal Medicine, Kansas University Medical Center, Kansas City, Kansas; ‡ Division of Cardiology, Kansas City Heart Rhythm Institute, Overland Park, Kansas; § Division of Cardiovascular Medicine, University of California Davis, Sacramento, California; ¶ Department of Medicine, Wayne State University, Detroit, Michigan; ‖ Department of Internal Medicine, University of Oklahoma, Oklahoma City, Oklahoma; ∗∗ Division of Cardiovascular Medicine, Stanford University, Stanford, California

**Keywords:** Left atrial appendage occlusion, Intracranial bleeding, Outcomes, Mortality

## Abstract

**Background:**

Percutaneous left atrial appendage occlusion (LAAO) has proved to be a safer alternative for long-term anticoagulation; however, patients with a history of intracranial bleeding were excluded from large randomized clinical trials.

**Objective:**

The purpose of this study was to determine outcomes in atrial fibrillation (AF) patients with a history of intracranial bleeding undergoing percutaneous LAAO.

**Methods:**

National Inpatient Sample and International Classification of Diseases, Tenth Revision, codes were used to identify patients with AF who underwent LAAO during the years 2016−2020. Patients were stratified based on a history of intracranial bleeding vs not. The outcomes assessed in our study included complications, in-hospital mortality, and resource utilization.

**Result:**

A total of 89,300 LAAO device implantations were studied. Approximately 565 implantations (0.6%) occurred in patients with a history of intracranial bleed. History of intracranial bleeding was associated with a higher prevalence of overall complications and in-patient mortality in crude analysis. In the multivariate model adjusted for potential confounders, intracranial bleeding was found to be independently associated with in-patient mortality (adjusted odds ratio [aOR] 4.27; 95% confidence interval [CI] 1.68–10.82); overall complications (aOR 1.74; 95% CI 1.36–2.24); prolonged length of stay (aOR 2.38; 95% CI 1.95–2.92); and increased cost of hospitalization (aOR 1.28; 95% CI 1.08–1.52) after percutaneous LAAO device implantation.

**Conclusion:**

A history of intracranial bleeding was associated with adverse outcomes after percutaneous LAAO. These data, if proven in a large randomized study, can have important clinical consequences in terms of patient selection for LAAO devices.

**Key Findings**

▪ Approximately 565 left atrial appendage occlusion (LAAO) implantations (0.6%) occurred in patients with a history of intracranial bleeding.
▪ History of intracranial bleeding was associated with a higher prevalence of overall complications and in-patient mortality in crude analysis.
▪ In multivariate adjusted analysis, intracranial bleeding was associated with mortality and overall complications after LAAO device implantations.

## Introduction

Patients with atrial fibrillation (AF) are at higher risk for thromboembolic events, with stroke being the most feared of such outcomes. Stroke prevention is the cornerstone of AF management and is achieved by oral anticoagulation or left atrial appendage occlusion (LAAO) in patients who have contraindications to long-term anticoagulation.^1^ It is estimated that 90% of left atrial thrombi originate from the left atrial appendage^2^; thus, LAAO has emerged as a novel therapeutic alternative for preventing thromboembolic events in AF patients who have a higher risk of concomitant bleeding.^3^^,^^4^

The United States (US) Food and Drug Administration approved the use of the Watchman LAAO device (Boston Scientific, Marlborough, MA) in March 2015 for stroke prevention in selected populations, and these procedures are supported by current guidelines from the American Heart Association (AHA), Heart Rhythm Society (HRS), and European Society of Cardiology.^1^^,^^5^ Large randomized clinical trials have demonstrated the noninferiority of percutaneous LAAO compared to warfarin for ischemic stroke prevention^3^^,^^4^; however, patients with a history of intracranial bleeding were excluded. Additionally, there is paucity of real-world data on the outcomes of percutaneous LAAO in this high-risk group of patients. Therefore, we sought to analyze the association of previous intracranial bleeding with procedural risk and inpatient adverse events in AF patients undergoing percutaneous LAAO from a large, nationally representative, contemporary sample of the US population.

## Methods

Data from the National Inpatient Sample (NIS) from 2016 to 2020 were used for the present study. The NIS is made possible by a federal–state–industry partnership sponsored by the Agency for Healthcare Research and Quality. The NIS includes hospital information for >7 million hospital discharges annually, accounting for 20% of all discharges from nonfederal hospitals in all 50 states. It also provides discharge weights for the computation of disease outcomes and health care utilization.^6^ Institutional Review Board approval and informed consent were not required for this study because of the deidentified nature of the dataset. The NIS adheres to the 2013 Declaration of Helsinki for the conduct of human research.

Patients with AF who underwent LAAO from January 2016 to December 2020 were identified using the *International Classification of Diseases, Tenth Revision, Clinical Modification* (ICD-10-CM) code of 02L73DK (occlusion of left atrial appendage with intraluminal device, percutaneous approach). Patients younger than 18 years and those with missing demographic data were excluded. The predominant device used in all LAAO procedures in our dataset was the Watchman device (Boston Scientific) because the Amplatzer Amulet device (Abbott, Abbott Park, IL) was not approved by the Food and Drug Administration during the time frame of our study. The study cohort was stratified into 2 groups based on a history of intracranial bleeding using ICD-10 codes (I60, I61, and I62). These ICD codes are well validated for identification of intracranial bleeding from administrative claims-based datasets as they have shown sensitivity and positive predictive value of 98.6% and 88.6%, respectively.^7^

Baseline characteristics, comorbidities, procedural complications, and in-patient outcomes, including mortality (reported as a distinct categorical variable in the dataset), length of stay (LOS), and hospitalization costs, were compared in LAAO device recipients based on intracranial bleeding status. Primary outcomes were in-hospital adverse events, LOS, and hospitalization costs. In-hospital adverse events were identified using ICD-10-CM codes (Supplemental Table 1). We also analyzed the independent association of intracranial bleeding with outcomes of mortality, overall and major complications (defined as composite of cardiac arrest, postoperative stroke, transient ischemic attack, arterial embolism, ST-segment elevation myocardial infarction, non–ST-segment elevation myocardial infarction, major bleeding, pericardial effusion requiring intervention, and peripheral vascular complications), LOS >1 day, and median hospitalization costs >$24,752. For computing hospitalization costs, the cost-to-charge ratio files supplied by the Healthcare Cost and Utilization Project were applied to the total hospital charges and adjusted for inflation to December 2020.

### Statistical analysis

Descriptive statistics are given as frequency (percentage) for categorical variables and as median [interquartile range] for continuous variables. Baseline characteristics were compared using the Pearson χ^2^ test and Fisher exact test for categorical variables and the Kruskal-Wallis *H* test for continuous variables. For crude comparison of procedural complications and in-hospital outcomes among the study groups, the Pearson χ^2^ test was used. For assessment of the independent association of previous intracranial bleeding with outcomes including mortality, major and overall complications, LOS >1 day, and median hospitalization costs >$24,752, a single-step multivariable logistic regression model was used. Age, sex, race/ethnicity, CHA₂DS₂-VASc score, hospital size, and Elixhauser comorbidities (valvular disease, pulmonary circulation disease, peripheral vascular disease, paralysis, neurological disorders, chronic pulmonary disease, diabetes without complications, diabetes with chronic complications, hypothyroidism, hypertension, renal failure, liver disease, peptic ulcer, acquired immune deficiency syndrome, lymphoma, metastatic cancer, solid tumor without metastasis, collagen vascular disease, coagulopathy, obesity, weight loss, fluid, and electrolyte disorders, chronic blood loss anemia, deficiency anemia, alcohol abuse, drug abuse, psychoses, and depression) were used for adjustment. These comorbidities were identified *a priori* and have been used extensively in the earlier studies from the administrative datasets for computing the adjusted outcomes.^8^ *P* <.05 was considered significant. All statistical analyses were performed using Statistical Package for Social Sciences (SPSS) Version 26 (IBM Corp., Armonk, NY) and R Version 3.6. Because of the complex survey design of NIS, sample weights, strata, and clusters were applied to raw data to generate national estimates.

## Results

A total of 89,300 percutaneous LAAO device implantations were included in our study after applying the relevant exclusion criteria. Of these procedures, approximately 565 LAAO device implantations (0.6%) were performed in AF patients with a history of intracranial bleeding, and 88,735 LAAO device implantations (99.4%) were performed in AF patients without a history of intracranial bleeding. Baseline characteristics of the study population are given in Table 1. Patients with a history of intracranial bleeding were more likely to be male and had a history of coagulopathy, diabetes mellitus, hypertension, liver disease, and weight loss, whereas those without a history of intracranial bleeding had more anemia, congestive heart failure, chronic pulmonary disease, renal failure, peripheral vascular disease, valvular heart disease, and obesity.

**Table 1:** Baseline characteristics of the study population stratified based on history of intracranial bleeding

	No intracranial bleeding [88,735 (99.4)]	Intracranial bleeding [565 (0.6)]	*P* value
Age (y)	77 [71–82]	74.5 [69–81]	<.01
Female	37 035 (41.7)	210 (37.2)	<.01
Age (y)			
<65	6450 (7.3)	70 (12.4)	<.01
65–74	28,370 (32.0)	210 (37.2)	
≥75	53,915 (60.8)	285 (50.4)	
Race			
White	75,315 (87.7)	455 (81.2)	<.01
Black	3705 (4.3)	30 (5.4)	
Hispanic	4005 (4.7)	15 (2.7)	
Asian or Pacific Islander	1145 (1.3)	35 (6.2)	
Native American	295 (0.3)	NR	
Other	1400 (1.6)	25 (4.5)	<.01
Comorbidities			
Anemia	15,410 (17.4)	80 (14.2)	<.01
Congestive heart failure	31,500 (35.5)	175 (31.0)	<.01
Coagulopathy	3405 (3.8)	25 (4.4)	<.01
Chronic pulmonary disease	19,875 (22.4)	110 (19.5)	<.01
Diabetes	16,245 (18.3)	155 (27.4)	<.01
Renal failure	21,685 (24.4)	130 (23.0)	<.01
Hypertension	77,260 (87.1)	505 (89.4)	<.01
Liver disease	2385 (2.7)	20 (3.5)	<.01
Obesity	15,510 (17.5)	85 (15.0)	<.01
Peripheral vascular disorder	7830 (8.8)	45 (8.0)	<.01
Valvular disease	5060 (5.7)	30 (5.3)	<.01
Weight loss	320 (0.4)	15 (2.7)	<.01
Hospital location			
Rural	1970 (2.2)	15 (2.7)	<.01
Urban nonteaching	9130 (10.3)	25 (4.4)	
Urban teaching	77,635 (87.5)	525 (92.9)	
Hospital size			
Small	9260 (10.4)	45 (8.0)	<.01
Medium	21,140 (23.8)	110 (19.5)	
Large	58,335 (65.7)	410 (72.6)	
Median income quartile			
0–25	19,260 (22.0)	125 (22.5)	<.01
26–50	23,175 (26.5)	165 (29.7)	
51–75	24,020 (27.4)	150 (27.0)	
76–100	21,060 (24.1)	115 (20.7)	

Values are given as n (%) or median [interquartile range] unless otherwise indicated.

n < 11 data was not reported per Healthcare Cost and Utilization Project recommendations and mentioned as not reportable (NR) in the table.

**Figure 1:**
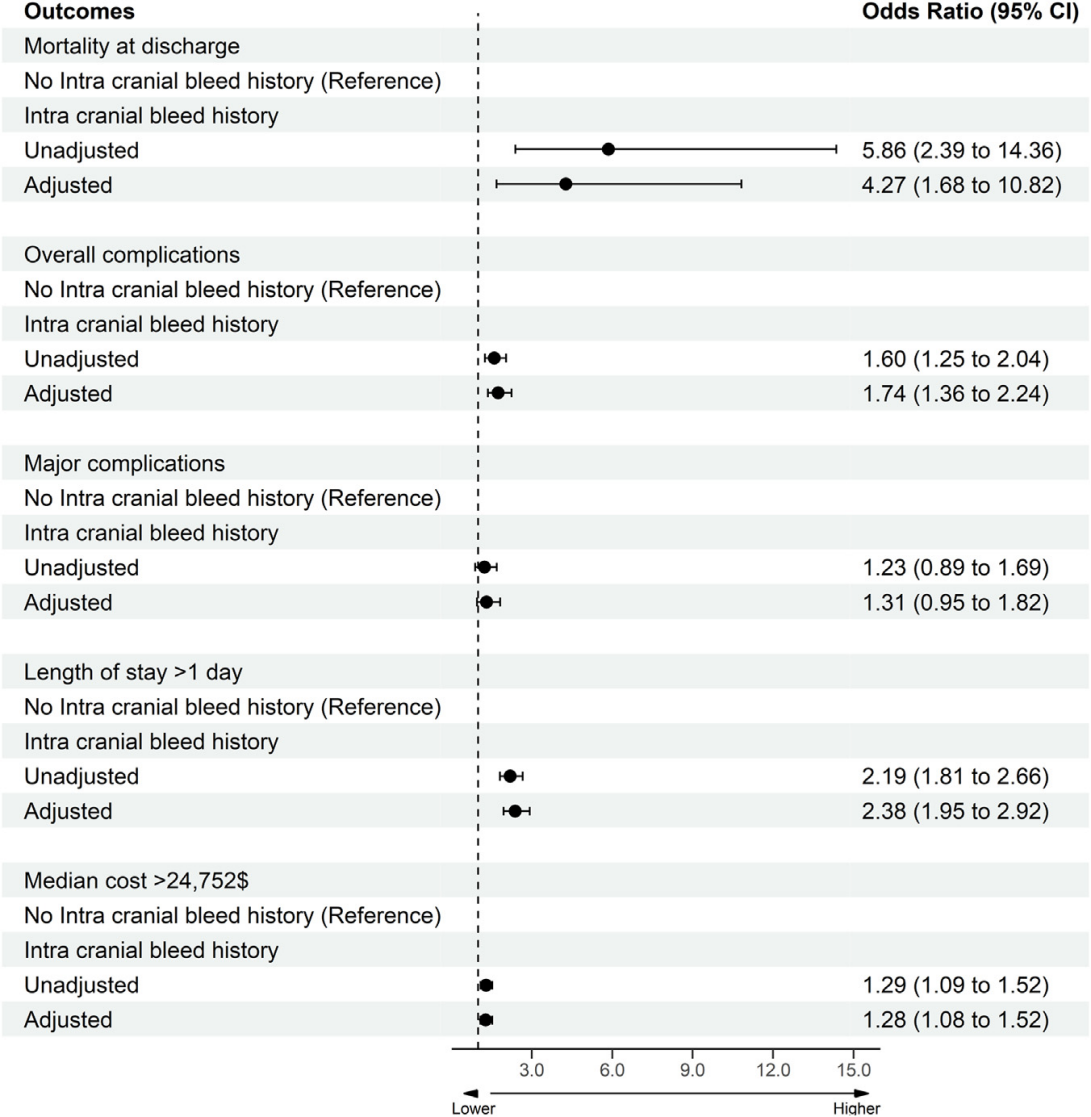
Unadjusted and adjusted association of history of intracranial bleeding with outcomes of mortality, overall complications, major complications, prolonged length of stay, and increased hospitalization costs in patients undergoing percutaneous left atrial appendage occlusion. CI = confidence interval.

Crude LAAO procedure-related complications stratified by history of intracranial bleeding status are given in Table 2. The prevalence of overall complications was higher in AF patients undergoing LAAO implantation with a history of intracranial bleeding compared to patients without a history of intracranial bleeding (13.3% vs 8.7%; *P* <.01). No difference in major complications between the 2 cohorts was identified (7.1% vs 5.9%; *P* = .2). The crude prevalence of any cardiovascular complication was similar between the 2 groups (2.7% and 2.7%, respectively; *P* = .92). The crude prevalence of any vascular complication was higher in patients with a history of intracranial bleeding compared to patients without a history of intracranial bleeding (6.2%, and 1.9%, respectively; *P* <.01). Crude inpatient outcomes after LAAO device implantation stratified by history of intracranial bleeding are given in Table 3. Patients with a history of intracranial bleeding had a higher prevalence of crude in-patient mortality than those without a history of intracranial bleeding (0.9% vs 0.2%, respectively; *P* <.01). The prevalence of non-home discharges after percutaneous LAAO was higher in patients with a history of intracranial bleeding than in patients without a history of intracranial bleeding (11.6%, and 2.4%, respectively; *P* <.01). To analyze the independent association of intracranial bleeding status with adverse outcomes, multivariable logistic regression models were created by adjusting for potential confounders (Figure 1). Intracranial bleeding was found to be independently associated with in-patient mortality (adjusted odds ratio [aOR] 4.27; 95% confidence interval [CI] 1.68–10.82); overall complications (aOR 1.74; 95% CI 1.36–2.24); prolonged LOS (aOR 2.38; 95% CI 1.95–2.92); and increased cost of hospitalization (aOR 1.28; 95% CI 1.08–1.52) after percutaneous LAAO device implantations.

**Table 2:** Crude left atrial appendage–related complications stratified based on history of intracranial bleeding

	No intracranial bleeding [88,735 (99.4)]	Intracranial bleeding [565 (0.6)]	*P* value
Overall complications	7750 (8.7)	75 (13.3)	<.01
Major complications∗	5195 (5.9)	40 (7.1)	.2
Any cardiovascular complication	2415 (2.7)	15 (2.7)	.92
Cardiac arrest	115 (0.1)	NR	NR
ST-segment elevation myocardial infarction	40 (0)	NR	NR
Non–ST-segment elevation myocardial infarction	1115 (1.3)	NR	NR
Pericardial effusion requiring intervention	875 (1.0)	15 (2.7)	<.01
Pericarditis	145 (0.2)	NR	NR
Cardiogenic shock	190 (0.2)	NR	NR
Any systemic complication	125 (0.1)	NR	NR
Anaphylaxis	25 (0.0)	NR	NR
Arterial embolism	65 (0.1)	NR	NR
Septic shock	40 (0.0)	NR	NR
Any peripheral vascular complication	1650 (1.9)	35 (6.2)	<.01
AV fistula	145 (0.2)	NR	NR
Pseudoaneurysm	285 (0.3)	NR	NR
Dissection	50 (0.1)	NR	NR
Postoperative stroke	50 (0.1)	NR	NR
Any hematologic complication	3390 (3.8)	30 (5.3)	<.01
Gastrointestinal bleeding	2095 (2.4)	NR	NR
Bleeding during the procedure	70 (0.1)	NR	NR
Need for blood transfusion	1395 (1.6)	25 (4.4)	<.01
Any pulmonary complication	2100 (2.4)	45 (8.0)	<.01
Respiratory failure	1005 (1.1)	25 (4.4)	<.01
Pneumothorax	25 (0.0)	NR	
Pleural effusion	310 (0.3)	15 (2.7)	<.01
Pneumonia	240 (0.3)	NR	NR
Long-term ventilation (>36 h)	90 (0.1)	NR	NR
Acute kidney injury	2145 (2.4)	15 (2.7)	<.01

Values are given as n (%) unless otherwise indicated.

n < 11 data was not reported per Healthcare Cost and Utilization Project recommendations and mentioned as not reportable (NR) in the table.

AV = arteriovenous.

∗ Composite of cardiac arrest, postoperative stroke, transient ischemic attack, arterial embolism, ST-segment elevation myocardial infarction, non–ST-segment elevation myocardial infarction, major bleeding, pericardial effusion requiring intervention, and peripheral vascular complications.

**Table 3:** Inpatient outcomes after left atrial appendage occlusion device implantation stratified based on history of intracranial bleeding

	No intracranial bleeding [88,735 (99.4)]	Intracranial bleeding [565 (0.6)]	*P* value
Died at discharge	135 (0.2)	5 (0.9)	<.01
Home/routine/self-care	86,455 (97.6)	495 (88.4)	<.01
Non-home discharges	2120 (2.4)	65 (11.6)	
Length of stay (d)	1 [1–1]	1 [1–1]	<.01
Cost of hospitalization ($)	25,140 [19,371–31,911]	26,786 [21,333–35,762]	<.01

Values are given as n (%) or median [interquartile range] unless otherwise indicated.

## Discussion

The main findings of our investigation are as follows. (1) The implantation rate of percutaneous LAAO in this nationally representative cohort of a US population with a history of intracranial bleeding was low at 0.6%. (2) Patients with a history of intracranial bleeding and LAAO device implantation had an increased prevalence of procedural complications in crude analysis. (3) Intracranial bleeding was independently associated with higher in-patient mortality, overall complications, prolonged LOS, and increased hospitalization costs after percutaneous LAAO device implantation.

AF patients with a history of intracranial bleeding represent a challenging cohort in terms of anticoagulation utilization, as some mechanisms of intracranial bleeding (spontaneous and lobar location likely due to underlying amyloid angiopathy) are associated with high recurrence rates and anticoagulation cannot be safely continued in such patients.^9^ In addition, intracranial bleeding in the setting of oral anticoagulation utilization has been associated with increased morbidity and mortality.^10^^,^^11^ Of note, this high-risk group also has an elevated risk of ischemic stroke.^12^ The landmark clinical trials comparing LAAO device implantation with warfarin have largely excluded patients with a history of intracranial bleeding.^3^^,^^4^ Our study is the first large analysis conducted to evaluate the outcomes of percutaneous LAAO in patients with prior intracranial bleeding and showed that such implantations were associated with increased mortality and overall complications. Additional studies with a specific focus on analysis of long-term outcomes after percutaneous LAAO implantation in patients with intracranial bleeding are needed to fully evaluate the safety and efficacy of these devices in such patients.

Few earlier studies have analyzed the association of intracranial bleeding with outcomes after percutaneous LAAO device implantation. Most of these studies were of single centers that have greater expertise in percutaneous LAAO device implantation, and our study was the first to report data from a national cohort that encompass the majority of LAAO implantations in the United States from 2016–2020. In a study of 198 patients with a history of intracranial bleeding, Tzikas et al^13^ found a higher prevalence of periprocedural major adverse events after LAAO device implantation; however, this effect did not reach statistical significance (5.4% vs 2.5%; *P* = .1). Similarly, in another study of 120 AF patients with previous intracranial bleed, Casu et al^14^ reported a higher rate of stroke (5.18%) and bleeding (6.62%) after LAAO device implantation. In a study of 63 patients with a history of intracranial bleeding, Hucker et al^15^ demonstrated that 95% of such patients did not experience a composite major endpoint of death, stroke, or major bleeding at 6 months of follow-up after LAAO device implantation. Of note, 19% of these patients were managed only with a dual antiplatelet strategy post-LAAO device implantation. In another study of 38 consecutive AF patients with previous intracranial bleed, Hutt et al^16^ found no strokes, intracranial bleeding, or deaths after percutaneous LAAO device implantation at 13 months of follow-up. All of their patients completed a standard 45-day course of anticoagulation post-LAAO implantation. Our dataset, unfortunately, does not inform on antiplatelet or anticoagulation strategy used after the implantation of a LAAO device. The CLEARANCE (Comparison of LAA-Closure vs Oral Anticoagulation in Patients With NVAF and Status Post Intracranial Bleeding; ClinicalTrials.gov Identifier: NCT04298723) randomized controlled clinical trial is currently recruiting participants and aims to compare outcomes in patients with prior intracranial bleed when stratified based on LAAO device implantation and oral anticoagulation and will provide further insights on the best treatment modality for stroke risk mitigation in these patients.^17^

### Study limitations

First, the NIS relies on ICD codes for disease and procedure identification, which may be subjected to errors; however, NIS uses a rigorous data quality control program to minimize miscoding and ensure data integrity.^6^ Second, long-term outcomes cannot be ascertained from the present dataset because NIS includes index admission data only. In addition, NIS does not provide information on the duration between intracranial bleeding and LAAO device implantation. Third, no data are available on procedural steps such as the amount of contrast used, duration of anesthesia, and operator experience. Fourth, NIS does not contain data on periprocedural antiplatelet or anticoagulation strategy, which can confound both short- and long-term outcomes after percutaneous LAAO device implantation. Fifth, NIS only caters to inpatient admissions and does not provide information on outpatient encounters. However, it should be noted that inpatient admission often is required for reimbursement of LAAO device implantation^18^; hence, our study constitutes a well-represented national sample of LAAO implantations in the United States in the contemporary period. Sixth, the ICD codes used to identify a history of intracranial bleeding may not be sensitive in stratifying hemorrhagic transformation of ischemic stroke,^7^ which may explain the lower incidence of intracranial bleeding in our cohort compared to the NCDR Left Atrial Appendage Occlusion Registry,^19^ as studies have shown that nearly one-third of patients with ischemic stroke have hemorrhagic transformation, and this phenomenon is especially associated with a history of AF and oral anticoagulant use.^20^

## Conclusion

Percutaneous LAAO in patients with a history of intracranial bleeding was associated with a higher risk of overall complication rates, greater in-hospital mortality, longer length of stay, and higher cost of hospitalization. Additional studies with long-term outcomes are needed to inform on the risks/benefits of LAAO device implantation in such patients.
