# Supplementary material for: Intracranial bleeding and associated outcomes in atrial fibrillation patients undergoing percutaneous left atrial appendage occlusion: Insights from National Inpatient Sample 2016–2020
Source: Heart Rhythm O2. 2023 Jun 8;4(7):433–9. doi: 10.1016/j.hroo.2023.06.002 (PMC10373143; doi:10.1016/j.hroo.2023.06.002)
Supplement: Supplementary Data [file mmc1.docx]

# ICD 10 codes

**Watchmen Device:**

02L73DK

Sequela for significant intracranial bleed

I60.1-2

1. **Cardiovascular:**
2. Cardiac Arrest/CPR procedure code: 5A12012
3. STEMI: I2101,02,03,09,i2111,i219,i212,i2121
4. NSTEMI or type II M: I21A,I21A1I21A9,I214
5. Air Embolism: T800XXA
6. Heart Failure: I5021,23,31,33,41,43
7. Heart Block complete: I442
8. Percutaneous coronary intervention: 0270, 0271,0272,0273
9. Mitral or aortic valve disorder: I34 and I35
10. Pericardial effusion/Hemopericardium: I31.2

10. Cardiac Tamponade: I314

11. Pericarditis: I300,I301,I308,I309

12. Need for Pericardiocentesis: 0W9D3, 0W9D4

13. Cardiogenic Shock: R570, T8111XA

14. Need for diagnostic left heart catheterization: B2100, B2101, B211, B212,B213,B215

**B. Systemic:**

15. Anaphylaxis T78

16. Arterial thrombosis: I74x

17. Deep venous thrombosis i82

18. Septic shock:R65.21

**C. Vascular complications:**

19. AV fistula: I300,I308,I309

20. Pseudoaneurysm: I72

21. Local site hematoma:M7981, L763

22. Local site bleeding: L760, L761, L762

23. Retroperitoneal Bleeding: K661

23. Dissection: I77.7, I71.0

**D. Neurologic:**

24. Hemorrhagic stroke: I60,I61,I62

25. Ischemic stroke: I63

26. TIA: G45

**E. Hematological complications:**

27. GI bleeding: K92

28. Hemothorax after procedure or unknown: J942

29. Need for blood products transfusion: 3023x

**F. Pulmonary:**

30. Post procedure or iatrogenic Pneumothorax and airleak: J93.5

31. Pleural Effusion: J90,J918

32. Pneumonia bacterial: J13-J18

33. Pulmonary embolism: I26

34. Respiratory failure: J960,J962,J969

**G. Kidney Injury**

35. Hemodialysis procedure code:5A1D70Z, 5A1D80Z, 5A1D90Z, 5A1D00Z, 5A1D60Z

36. AKI: N17
